# Validation of the Vectra XT three-dimensional imaging system for measuring breast volume and symmetry following oncological reconstruction

**DOI:** 10.1007/s10549-018-4843-6

**Published:** 2018-06-05

**Authors:** Rachel L. O’Connell, Komel Khabra, Jeffrey C. Bamber, Nandita deSouza, Farid Meybodi, Peter A. Barry, Jennifer E. Rusby

**Affiliations:** 1 Department of Breast Surgery, The Royal Marsden NHS Foundation Trust, Downs Road, Sutton, Surrey SM2 5PT UK; 2 Department of Statistics, The Royal Marsden NHS Foundation Trust, Sutton, UK; 3 Joint Department of Physics and Cancer Research UK Cancer Imaging Centre, Institute of Cancer Research and Royal Marsden NHS Foundation Trust, Sutton, UK; 4 Cancer Research UK Cancer Imaging Centre, Institute of Cancer Research and Royal Marsden NHS Foundation Trust, Sutton, UK; 5 Westmead Breast Cancer Institute, Westmead, NSW 2145 Australia

**Keywords:** Breast cancer, Breast volume, Aesthetic outcome, Breast conservation, Breast surgery

## Abstract

**Purpose:**

Three-dimensional surface imaging (3D-SI) of the breasts enables the measurement of breast volume and shape symmetry. If these measurements were sufficiently accurate and repeatable, they could be used in planning oncological breast surgery and as an objective measure of aesthetic outcome. The aim of this study was to validate the measurements of breast volume and symmetry provided by the Vectra XT imaging system.

**Methods:**

To validate measurements, breast phantom models of true volume between 100 and 1000 cm^3^ were constructed and varying amounts removed to mimic breast tissue ‘resections’. The volumes of the phantoms were measured using 3D-SI by two observers and compared to a gold standard. For intra-observer repeatability and inter-observer reproducibility in vivo, 16 patients who had undergone oncological breast surgery had breast volume and symmetry measured three times by two observers.

**Results:**

A mean relative difference of 2.17 and 2.28% for observer 1 and 2 respectively was seen in the phantom measurements compared to the gold standard (*n* = 45, Bland Altman agreement). Intra-observer variation over ten repeated measurements demonstrated mean coefficients of variation (CV) of 0.58 and 0.49%, respectively. The inter-observer variation demonstrated a mean relative difference of 0.11% between the two observers. In patients, intra-observer variation over three repeated volume measurements for each observer was 3.9 and 3.8% (mean CV); the mean relative difference between observers was 5.78%. For three repeated shape symmetry measurements using RMS projection difference between the two breasts, the intra-observer variations were 8 and 14% (mean CV), the mean relative difference between observers was 0.43 mm for average symmetry values that ranged from about 3.5 to 15.5 mm.

**Conclusion:**

This first validation of breast volume and shape symmetry measurements using the Vectra XT 3D-SI system suggests that these measurements have the potential to assist in pre-operative planning and also as a measure of aesthetic outcome.

## Introduction

Three-dimensional surface imaging (3D-SI), first described by Burke and Beard [1, 2] in 1967 to analyse facial structures, is now also used in the aesthetic breast surgery setting, mainly as a marketing tool. Patients can visualize three-dimensional (3D) images of themselves and their plastic surgeon can manipulate these images to simulate the expected appearances after breast augmentation or reduction. Clinicians and researchers can also exploit 3D-SI to measure breast volume and symmetry in pre-operative planning, to investigate volume retention after lipofilling and use it as a measure of cosmetic outcome after surgery [3]. Moreover, the ability to measure the shape symmetry, which reports not only on the volume but also on the surface shape, is advantageous [4]. By reflecting the mirror image of one of the patient’s breasts onto the other, the distance between the two breast surfaces may be calculated over the entire surface of the breast from the superimposed images. However, 3D-SI has not hitherto been exploited in a breast cancer population. In these patients, where body mass index is higher [5] compared to the slim build of the aesthetic breast augmentation population [6], 3D-SI may not give accurate volume measurements as it requires an assumption about the depth and curvature of the underlying chest wall. Thus, the validity of the volume and symmetry measurements in this population remains unknown and its use as a clinical tool is not established. The aim of this study, therefore, was to validate breast volume measurement using the Vectra XT (Canfield Sci, New Jersey, USA), a popular 3D imaging system used worldwide, and to investigate the reproducibility of measuring breast volume and symmetry in a breast cancer population who had undergone oncological breast surgery.

## Methods

### Phantom model evaluation

**Fig. 1:**
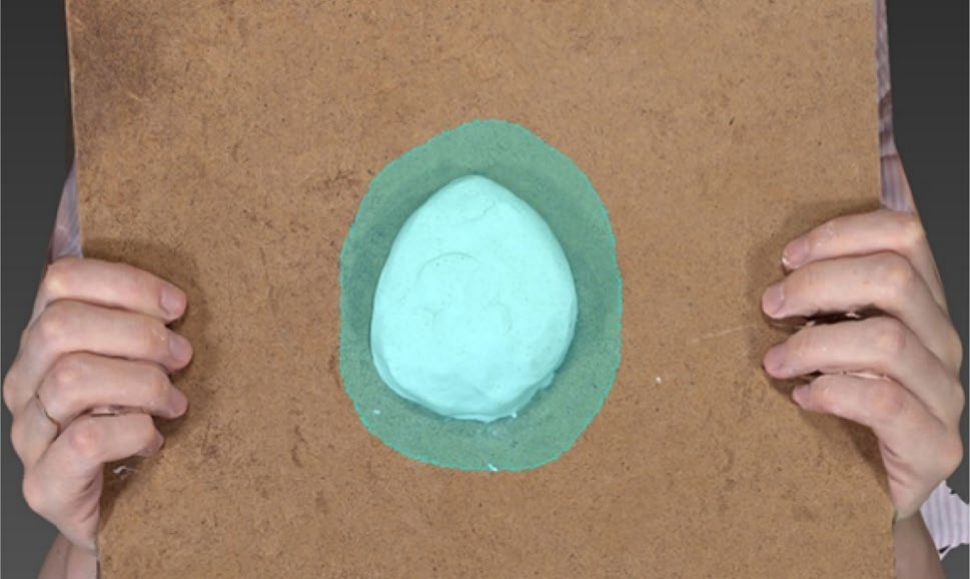
Image of breast phantom made of plasticine taken using the Vectra XT camera. The observer has identified a region of interest around the perimeter of the phantom that includes an area 2 cm beyond the boundary of the model

The breast phantoms were made of plasticine (NCP Newclay Products, Devon, UK). Six phantoms were constructed, each of a different volume (Fig. 1), incorporating a wide range of breast volumes. ‘Resections’ were sequentially excised from each phantom and after each resection the phantom was re-imaged. The resections were used to represent typical excision biopsies, wide local excisions and therapeutic mammoplasties. In total, 45 phantoms were imaged with the following starting volumes (number of resections in parentheses): 100 (5), 200 (5), 300 (5), 400 (5), 500 (9) and 1000 (10) cm^3^. The gold standard volume of each phantom was measured initially and after every resection using the Archimedes’ principle of water displacement. The phantoms were placed on a wooden board held 1.5 metres from, and parallel to, the Vectra XT. 3D-SI of the phantoms initially and after every resection was carried out using a standard protocol available in the Vectra Analysis Module (VAM) software (Canfield Sci, New Jersey, USA). By identifying a region of interest (ROI) including an area bounded by a 2-cm perimeter drawn on the 3D image beyond the boundary of the phantom, the posterior wall of the phantom was interpolated from the ROI data and the volume enclosed by the breast phantom and its estimated posterior wall was calculated. Two observers independently measured the volume for each phantom 10 times, from which the mean volume and coefficient of variation (CV) were calculated.

The volume measurement accuracy was obtained by comparing the mean volumes for each phantom measured by 3D-SI to the gold standard. Agreement between the two methods was determined using Bland Altman plots [7] displaying the relative difference between the mean of the ten estimates and the gold standard volume ((mean 3D-SI volume − gold standard volume)/gold standard × 100) as a function of the gold standard volume, and ‘limits of agreement’ (overall mean relative difference +/− 1.96SD of the mean relative difference).

To assess intra-observer variation, the coefficient of variation (CV = SD/mean) for the 10 measurements of each of phantom was calculated and then the mean of the CV was calculated as an indication of the variation in repeated measurements for all of the phantoms.

Inter-observer agreement was measured using a Bland Altman plot showing the relative difference between the means of ten estimates for each observer ((mean volume observer 1 − mean volume observer 2)/(mean of mean volumes for observer 1 and 2) × 100) as a function of the mean of the two observers’ mean volume measurements, and ‘limits of agreement’ (overall mean relative difference +/− 1.96 SD of the mean relative difference).

### In vivo patient evaluations

Following Regional Ethics Committee approval (REC number 14/YH/0175) 16 patients were recruited to the study. Informed consent was obtained from all individual participants included in the study. Additional informed consent was obtained from the participant whose images are included in this article. Participants were positioned 1.5 m away from the Vectra XT. The participant’s face was excluded from all images. Participants were imaged with their hands on their hips at the end of normal inspiration.

### Measurement of volume

**Fig. 2:**
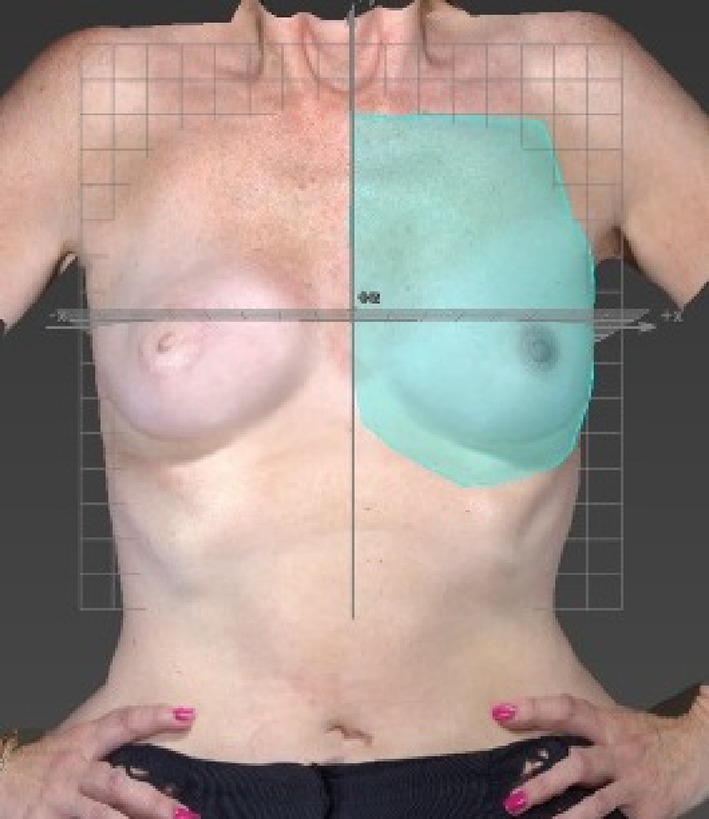
Method developed to measure volume using Vectra-XT 3D-SI

After attempting to measure volume using simple placement of landmarks and the in-built software, and secondly by simply marking an ROI by eye, we concluded that there was too much variation and this had an unacceptable impact on reproducibility. Therefore to calculate the breast volume using the VAM software (Canfield Sci, New Jersey, USA), a specific protocol was developed (Fig. 2). A grid was placed on the image of the breast in the *x, y, z* planes, with each grid cube having a side of 2 cm. The y axis was positioned at the midline, with the upper border of the grid at the suprasternal notch. A region of interest (ROI) was identified around the perimeter of the breast as follows:

1. Vertically down the midline from one square below the suprasternal notch to one square below the most medial aspect of the infra-mammary fold (IMF).
2. Then moving laterally at a level one square below the IMF to the anterior axillary line following parallel to the curve of the IMF or ptosis of breast (whichever is more caudal).
3. Then moving superiorly along the anterior axillary line towards the axilla.
4. Then moving medially to define the superior border to meet the midline one square below the clavicle.

The volume measurements were undertaken by two observers imaging each patient three times. The left and right breasts were treated independently.

To assess intra-observer variation, the mean CV for all breasts was calculated. Bland Altman plots were used to measure inter-observer variation and agreement as described for the phantom studies.

### Measurement of shape symmetry

**Fig. 3:**
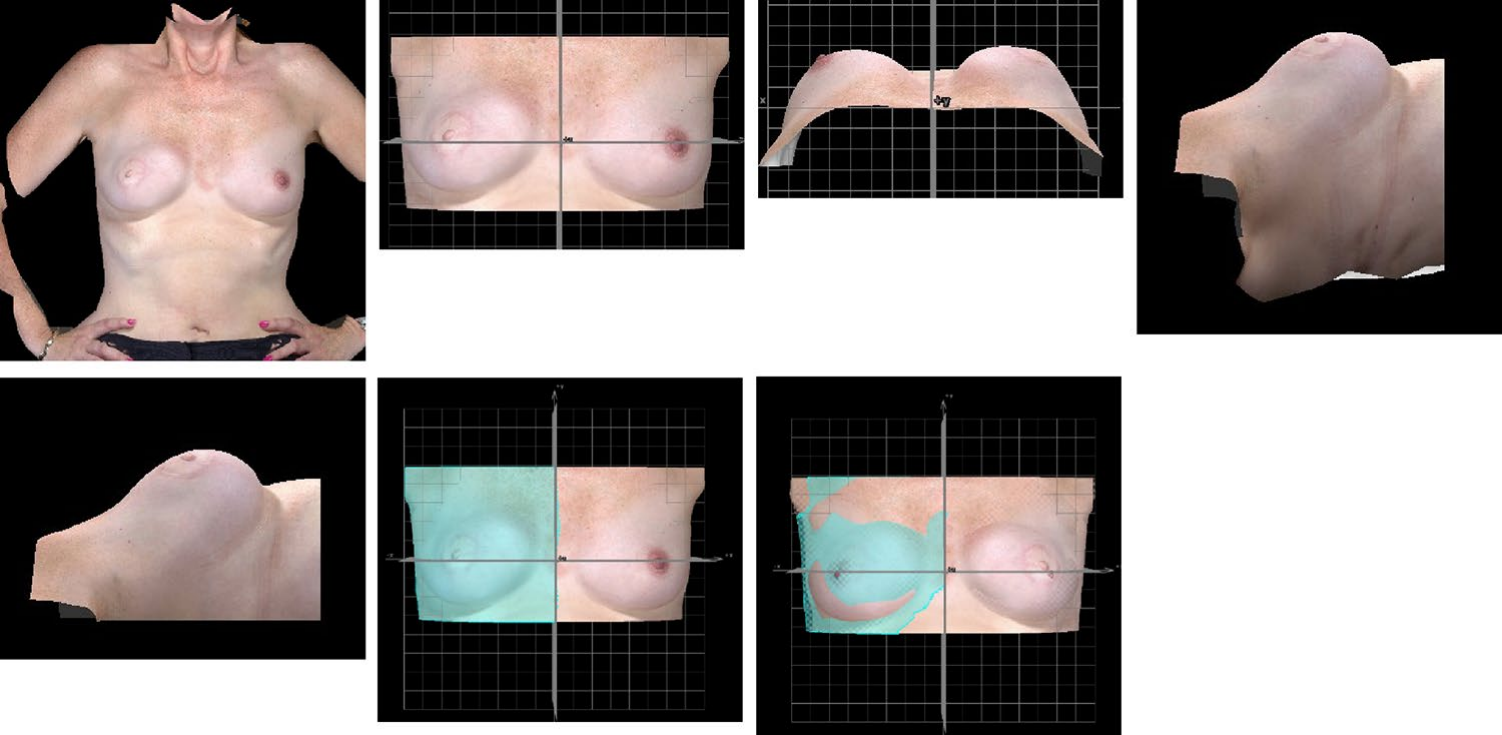
Protocol to measure breast symmetry, RMS Projection difference (RMS-PD). **a** AP view of torso, **b** gridlines placed onto image and cropped, **c** torso in craniocaudal view, **d** image rotated laterally, **e** image cropped at level of the anterior axillary line, **f** one-half of torso is selected, Image is copied and reflected in *x* = 0

Similarly, a protocol was developed to measure symmetry. For each 3D image:

1. The torso was rotated to anterior-posterior view (AP) view (Fig. 3a).
2. The gridlines were place onto the image so that the y axis (*x* = 0) bisected the torso. The image was cropped so that only the breast area was visible. (Fig. 3b).
3. The torso was viewed cranio-caudally and the shoulders/clavicles aligned along the line of *z* = 0 in case the patient was not standing parallel to the Vectra XT cameras at the time of imaging (Fig. 3c).
4. The image was rotated 90° laterally and cropped either side at the level of the anterior axillary line (Fig. 3d, e).
5. The image was returned to the AP position. One-half of the torso was selected from the *y* axis laterally using a lasso tool provided within the VAM software (Fig. 3f).
6. The whole image was copied and reflected with the y axis as the line of symmetry, inverting the *z*-axis data so that in both the original and the reflected images the z position of the breast surface was positive. Using the VAM software, the distances along the *z* axis (skin surface ‘height’) between the corresponding *x*–*y* coordinate points on the original image and the reflected image were measured. (Fig. 3g). This measurement was termed the height difference (HD). The degree of asymmetry was then quantified using the RMS projection difference (RMS-PD), as the square root of the mean of the squared HDs, which gives a positive value regardless of whether left is larger than right or vice versa. The lower the number, the more symmetrical the breasts is.

Both observers calculated the RMS-PD for each of the three images of each patient. To assess intra-observer variation, the mean of the CV was calculated. A Bland Altman plot was used to measure inter-observer agreement and variation as above.

Data were entered into an excel spread sheet (Microsoft Corp., Redmond, Washington) and analysed using SPSS statistical software (SPSS v22; SPSS, Inc., Chicago).

## Results

### Phantom model evaluations

#### Accuracy

**Fig. 4:**
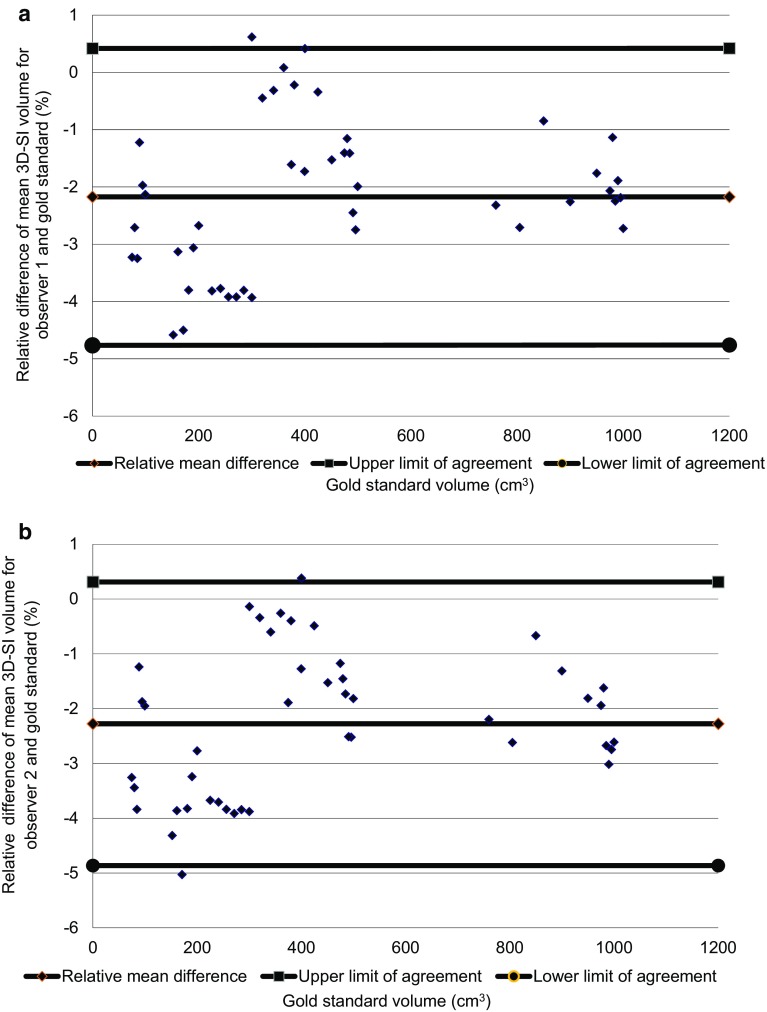
**a** Bland Altman plot of agreement between gold standard and mean Vectra XT for Observer 1 for phantom breast model volumes, **b** Bland Altman plot of agreement between gold standard and mean Vectra XT for observer 2 for phantom breast model volumes

The mean relative differences for observers 1 and 2 compared with the gold standard were 2.17 and 2.28% with limits of agreement of − 0.42 to 4.76% and − 0.33 to 4.95%, respectively (Fig. 4a, b).

#### Intra-observer variability

The mean CVs were 0.58 and 0.49% for observers 1 and 2, respectively. No significant correlation was seen between CV and volume (correlation coefficients 0.026 and 0.273, and *p* values 0.867 and 0.069, respectively, for observer 1 and 2), thus justifying the averaging of CV over phantoms of different volumes.

#### Inter-observer agreement and variability

**Fig. 5:**
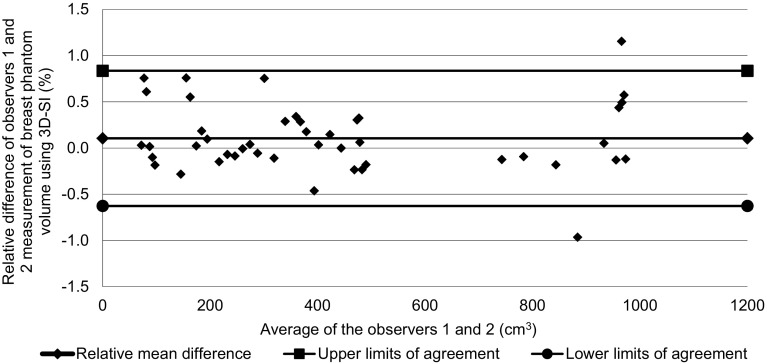
Bland Altman plot of agreement between observers 1 and 2 for phantom breast model volumes

The Bland Altman plot that compared one observer’s volume measurements with the other’s (Fig. 5) demonstrated a mean relative difference for 3D-SI volume measurements between the two observers of 0.11% and limits of agreement of − 0.63 to 0.84%.

#### Repeatability and reproducibility of volume and shape symmetry measurements in patients

Of the 18 women invited to participate in this validation study, 16 agreed. The other two were unable to attend in the allocated timeframe. Eleven women had undergone mastectomy and reconstruction and 5 had undergone breast conservation. The average age was 53 years (SD = 9 years) and the average BMI was 24.8 kg/m^2^ (SD = 4.9 kg/m^2^).

#### Volume measurements

The mean CV over three repeated volume measurements was 3.9% and 3.8% for observer 1 and 2, respectively. No significant correlation was observed between CV and breast volume (correlation coefficients 0.025 and − 0.17, and *p* values 0.891 and 0.366, respectively, for observer 1 and 2), thus justifying the averaging of CV over breast volume.

**Fig. 6:**
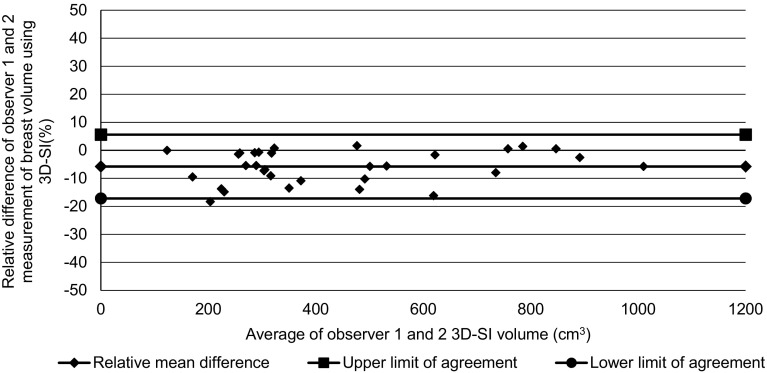
Bland Altman plot of agreement between observers 1 and 2 for patients’ breast volumes

The Bland Altman plot (Fig. 6) demonstrated a mean relative difference for 3D-SI volume between the two observers of − 5.78% with limits of agreement of − 17.76 to 6.2% for patients’ breast volume measurements obtained by averaging over three estimates.

#### Shape symmetry measurements

The mean CV of repeated volume measurements was 8.0 and 14.0% for observer 1 and 2, respectively.

**Fig. 7:**
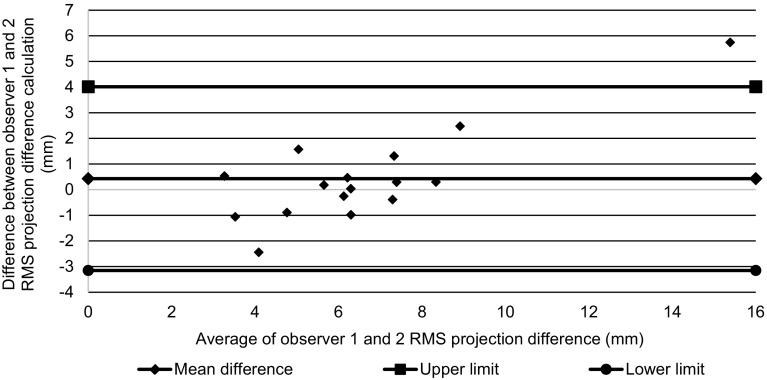
Bland Altman plot of agreement between observers 1 and 2 for breast symmetry

The Bland Altman plot (Fig. 7) demonstrated that the mean difference between the observers’ estimates of symmetry was 0.43 mm, for average symmetry values that ranged from about 3.5 mm to about 15.5 mm, with upper limit of agreement of 4.01 mm and lower limit of agreement of − 3.15 mm.

## Discussion

We have demonstrated the ability to assess the volume and shape symmetry of breasts in a breast cancer population with prior oncological surgery and shown a high degree of reproducibility between observers. Invasive breast cancer affects approximately 55,000 women a year in the United Kingdom [8] and at least 80% have some form of surgery [9] that necessitates the best possible aesthetic outcome because it influences psychological recovery and quality of life [10–12]. The ability to objectively measure the volume and symmetry of the breasts has the potential to profoundly influence surgical planning and thus aesthetic outcome.

As was shown in Fig. 4a, b, the study demonstrated that the Vectra XT is able to measure the volume of a simple breast-shaped object with an average accuracy of about 2.2% underestimation of the true volume (one observer − 2.17%, the other − 2.28%). Variation in accuracy over the volume range studied suggested that most of this underestimation occurred at volumes smaller than 300 cm^3^, and for larger volumes there may even have been a slight overestimation. These trends were consistent across the two observers. The intra-observer variation was found to be low (mean CV 0.58% over ten estimates for one observer and 0.49% for the other). The two observers strongly agreed; inter-observer agreement obtained from the Bland Altman plot (Fig. 5) was on average within 0.11% of the volume measured i.e. there was no significant bias of one observer relative to the other compared with the residual SD of 0.38% seen in Fig. 5.

A similar study of a 3D-SI system designed and made in-house by researchers at the University of Glasgow was validated using a phantom of the breast [13]. In their study, the authors found that on average, relative to the gold standard, their 3D-SI volume measurements overestimated the volumes by 5%, whereas in this study the bias was slightly smaller at about 2% underestimation. Another study using phantoms to validate the Minolta Vivid 910 (Konica Minolta Co Ltd, Osaka, Japan) 3D-SI system was undertaken by Kovacs et al. [14] who used ‘dummy models’ of the breasts consisting of two mannequins. The authors found that there was a lower variance (CV) for repeatability of breast volume measurements performed by an experienced observer compared to those with less experience (lowest CV 1.28%, highest 2.5%). They related this to the ability of the observers to accurately mark the breast borders on the thoracic wall as per a defined and standardised protocol. In our experiment using the plasticine phantoms, the boundaries of the phantom were easily identifiable, which explains why our intra- and inter-observer variabilities were low.

The second part of the present study aimed to test the intra-observer and inter-observer variation of human breast volume and symmetry measurements. The intra-observer variation in the human breasts was on average 3.9% for one observer and 3.8% for the other, which are much larger than the values obtained using the phantoms (around 0.5%). This is likely to be due to both the complexity of defining the region of interest in human breasts compared to the simple outline of the phantoms, and the fact that only three estimates were made in each human breast whereas ten estimates were obtained of each phantom’s volume. We chose to use three measurements of the breast volume as in clinical practice it would not be practical to measure the breasts more than several times due to time constraints. The mean relative difference for volume measurements between the two observers was 5.78%, which was smaller than the residual SD of 6.1% on this Bland Altman plot but probably significant in terms of a bias of one observer compared with the other. Cochrane et al. [15] identified that excision of greater than 10% of the breast volume led to a reduction in patient satisfaction. In addition, Sigurdson et al. [16] showed that the minimum volume difference detectable by the human eye was 50 cm^3^ by subjective judgment. Therefore, we assert that a relative mean difference of 5.78%, which was maintained for breasts volumes up to 1000 cm^3^ and thus equal to about 58 cm^3^ at the largest volume, is adequate.

Mailey et al. [17] conducted a study in 22 patients undergoing breast augmentation whereby two observers measured the breast volumes before and after augmentation to investigate the accuracy of measuring breast and implant volume. They used the Portrait 3D surgical simulation platform (Axis Three, Boston, Massachusetts, USA). The authors found no significant difference between their two observers’ estimations of breast volumes pre- or post-operatively, and high reliability (intra-class correlation coefficient, ICC all > 0.94). In another study, Losken et al. [18] measured the volume of 19 breasts of patients using the 3dMD system (3dMD LLC, Atlanta, Georgia, USA) before they underwent mastectomy and used water displacement of the mastectomy specimen as the gold standard. They concluded that intra-observer reliability was excellent with an ICC of 0.975 and inter-observer reliability was also excellent with an inter-class correlation coefficient of 0.968. However, no quantitative measurements of repeatability or reproducibility were provided.

The RMS-PD or similar methodology has been used as a measure of shape symmetry in other studies [19–23]. In our study, population RMS-PD ranged between 3 and 16 mm (see Fig. 7). Another study [24] found that the average symmetry score measured using RMS-PD had a range of 1.7–12.8 mm in a cohort of 87 women without a history of breast surgery. It was significantly higher in patients with a higher BMI, cup size, and chest wall circumference. As expected, the RMS-PD values in our study were higher since they had all undergone surgery.

An objective symmetry score has the potential to be used for measurement of aesthetic outcome after surgery. It is important, however, to understand what repeatability and reproducibility are necessary for a symmetry score to be useful in this way. A minimum requirement is that the score is significantly different for different subjective assessments of aesthetic outcome. One study [20] compared objective symmetry scores of this type with subjective scores for symmetry in forty-four patients after immediate unilateral breast reconstruction with extended latissimus dorsi flap. There was a highly significant correlation (*p* < 0.0001, correlation coefficient = 0.62) between the two scores. We also recently published a study demonstrating that RMS-PD symmetry significantly agrees (Kruskal Wallis test, *p* < 0.001) with panel assessment of aesthetic outcome in women who have undergone breast conserving therapy [25]. In that study, patients were grouped according to panel assessment of the aesthetic outcome after surgery (poor/fair/good/excellent). The median RMS-PD scores for each group differed by approximately 2 mm from those in the next sequential group. Therefore, the repeatability (intra-observer highest CV < 14%, highest symmetry score in patient 15.5 mm, therefore worst SD 2.2 mm) and reproducibility (inter-observer mean relative difference < 0.5 mm) measured here should be sufficient for the method to have clinical value.

This study has demonstrated satisfactory repeatability and reproducibility of breast volume and symmetry measurements using the Vectra XT three-dimensional surface imaging system. However, these data provide preliminary results in a small cohort of patients. Before widespread clinical use, further validation should be undertaken by independent breast surgical research groups in a larger cohort of patients with a wide variety of body habitus, and who have undergone a variety of breast surgery.

## Conclusion

We are the first to validate breast volume and symmetry measurement using the popular Vectra XT 3D-SI, which is essential before widespread use. For volume measurement, the intra-observer variation was low (CV < 4%), and mean difference between the two observers was acceptable at about 5.8%. For symmetry of the breasts, the intra-observer variation was low (CV < 14%) and mean inter-observer difference was 0.43 mm in a study population for which the range of symmetry values was 3–16 mm. This repeatability and reproducibility would be adequate for clinical use in pre-operative planning of breast surgery and as measures of aesthetic outcome after breast cancer surgery. However, further validation is required before widespread clinical use.
